# Evidence-based occupational health

**DOI:** 10.47626/1679-4435-2023-212

**Published:** 2023-08-08

**Authors:** Wanderley Marques Bernardo

**Affiliations:** Professor of Medicina Baseada em Evidências at Faculdade de Medicina da Universidade de São Paulo; coordinator of Programa Diretrizes da Associação Médica Brasileira

Worldwide, there still exists an insistent and uncomfortable posture of ignoring or, worse, distorting the scientific basis of health care actions. And decision-making in occupational medicine is no different.

Obviously, this posture is not accidental, involuntary or purposeless; it is replete with political, economic, legal and power interests, which rightly fear any patient-centered evidence-based initiative.

But, despite such constant pressure, there are initiatives to confront these spurious interests by redirecting our actions towards benefits and safety, based on quality clinical research that is relevant, transparent, and involves important outcomes for patients.

One such initiative is ongoing in the National Association of Occupational Medicine (ANAMT), which trains its leadership in the main concepts of patient-centered evidence-based practice. This knowledge can be then multiplied among peers, resulting in further evidence-based clinical guidelines.

More than 10 occupational physicians have spontaneously dedicated themselves to researching occupational issues such as biological risk, heat, cold, mental health, vibration, sound, long COVID, remote work, and diagnosis. They are sharing their findings in bimonthly training meetings and constant mentoring, addressing topics such as how to structure clinical questions, searching for and selecting evidence, the critical evaluation and synthesis of evidence, and the recommendations and main components of evidence-based guidelines.

The foundations and importance of scientific evidence in the life of humanity cannot be summarized here, nor can we further elaborate on the selfishness and greed standing in opposition to the good, which we, as doctors and health professionals, strive for in our work. However, ANAMT provides 3 further questions for reflection, especially in the wake of the conduct and management atrocities witnessed during the COVID-19 pandemic:

- Whose role is it to define effective and safe options in shared decision-making between doctors and patients?
- What is the role of scientific evidence in this definition, and why do physicians and health professionals need to understand it?
- Can we really imagine the existence of institutions that attempt to advance human life by applying evidence but are themselves immune to the range of conflicts of interest experienced by health systems throughout the world?

Regardless of whether we consider the idea of thinking about each other utopian, ANAMT’s initiative to develop worker-centered evidence-based guidelines should be praised.

